# Supplementary material for: Orientationally Averaged Version of the Rotne–Prager–Yamakawa Tensor Provides a Fast but Still Accurate Treatment of Hydrodynamic Interactions in Brownian Dynamics Simulations of Biological Macromolecules
Source: J Chem Theory Comput. 2023 Jul 6;19(15):5099–111. doi: 10.1021/acs.jctc.3c00476 (PMC10413861; doi:10.1021/acs.jctc.3c00476)
Supplement: Supplementary file 1 — ct3c00476_si_001.pdf [file ct3c00476_si_001.pdf]

Supporting Information for:

An Orientationally Averaged Version of the Rotne-Prager-Yamakawa Tensor Provides A Fast But Still Accurate Treatment Of Hydrodynamic Interactions In Brownian Dynamics Simulations Of Biological Macromolecules

John W. Tworek[a] & Adrian H. Elcock[a*]

[a]Department of Biochemistry & Molecular Biology, University of Iowa, Iowa City, IA 52242, United States of America.

*email: adrian-elcock@uiowa.edu

The eleven proteins studied here are: B1 immunoglobulin-binding domains of protein G (Protein G), B1 immunoglobulin-binding domain of protein L (Protein L), chymotrypsin inhibitor 2 (CI2), barnase, fyn SH3 domain (fyn-SH3), cold shock protein B (CSPB), intestinal fatty acid binding protein (IFABP), Semliki Forest viral capsid protein (SFVP), λ-repressor, colicin E9 immunity protein (IM9), and apo-calmodulin (apoCaM). The six RNAs studied here are: *S. cerevisiae* $tRNA^{Phe}$, Turnip yellow mosaic virus (TYMV) tRNA-like structure, *E. coli* 5S rRNA, *T. thermophilus* P4-P6, *B. subtilis* mgtE5' UTR sequence, and an 85 nucleotide stem loop. The RCSB (https://www.rcsb.org) IDs of the structures used to build the simulation models for these molecules are listed, along with their total residue counts, in Table S1.

**Molecule**	**RCSB ID**	**Nres**
protein G	1PGA	56
protein L[a]	1HZ6	64
CI2	2CI2	65
Barnase	1BNI	108
fyn-SH3	1SHF	59
CSPB	1CSP	67
IFABP	1IFC	131
SFVP	1VCP	149
λ-repressor[b]	1LMB	80
Im9	1IMQ	86
apo-CaM[c]	1CFD	148
S. cerevisiae $tRNA^{Phe}$	1EHZ	76
TYMV tRNA-like structure	4P5J	85
85 nt Stem Loop[d]	N/A	85
E. coli 5S rRNA	1C2X	120
T. thermophilus P4-P6	1GID	158
B. subtilis mgtE 5′ UTR	3PDR	161

Footnotes:

[a] Y47W mutant.

[b] Residues 6-85

[c] Averaged NMR structure

[d] Model created in house (Henderson et al. 2023)

Table S1

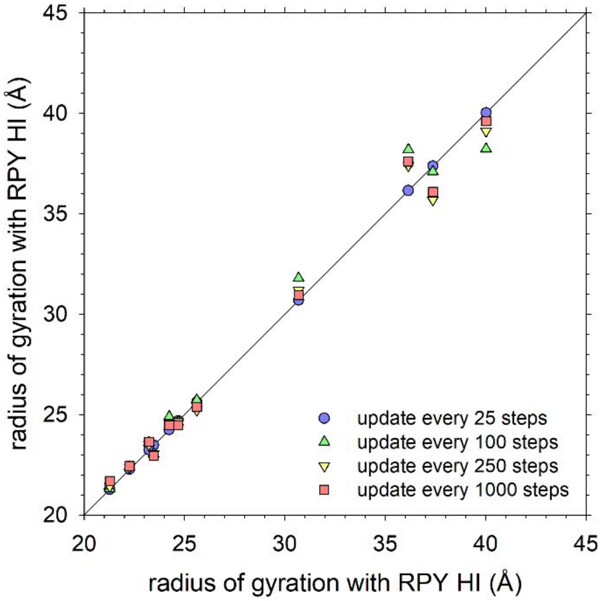

Figure S1 Comparison of the mean radius of gyration values obtained from simulations of unfolded proteins performed with the full RPY model for different update intervals for the diffusion tensor with those obtained with the full RPY model with an update interval of 25 steps. The solid line represents the line y = x.

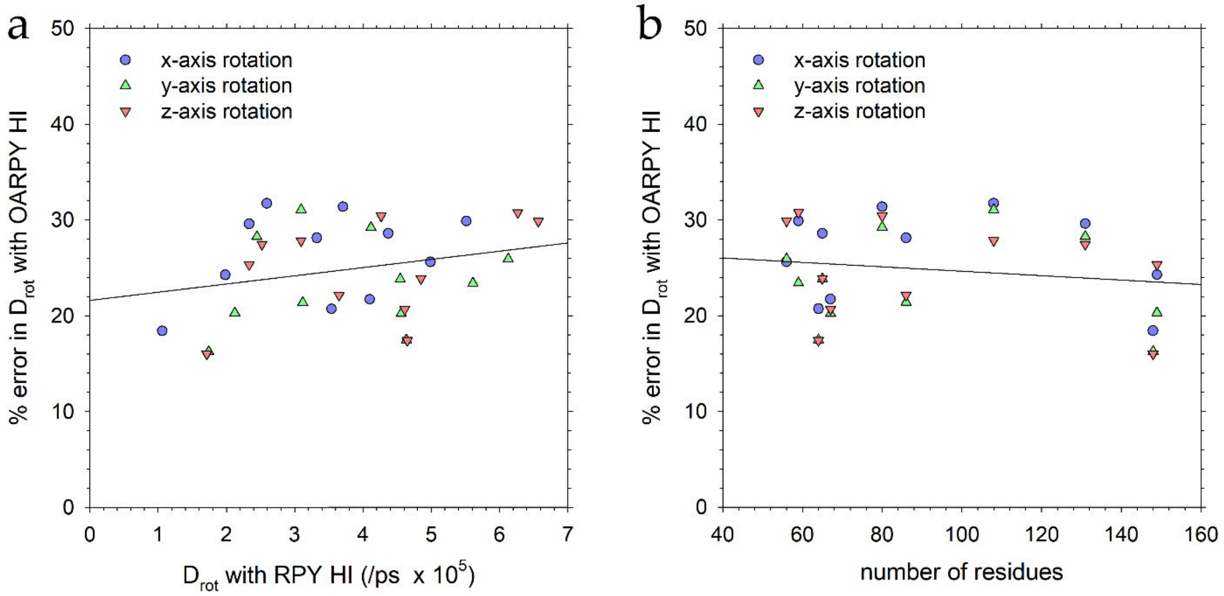

Figure S2 (a) Plot showing the percentage error in the rotational diffusion coefficient (D_{rot}) obtained from the OA RPY model (relative to the value obtained with the full RPY model) versus the D_{rot} value obtained from the full RPY model. Different symbols are used for rotation around each of the three principal axes. The r^2 value of the linear regression (black line) is 0.063. (b) Plot showing the same percentage error in the D_{rot} value shown in (a) but plotted versus the number of residues in the protein. The r^2 value of the linear regression (black line) is 0.023.

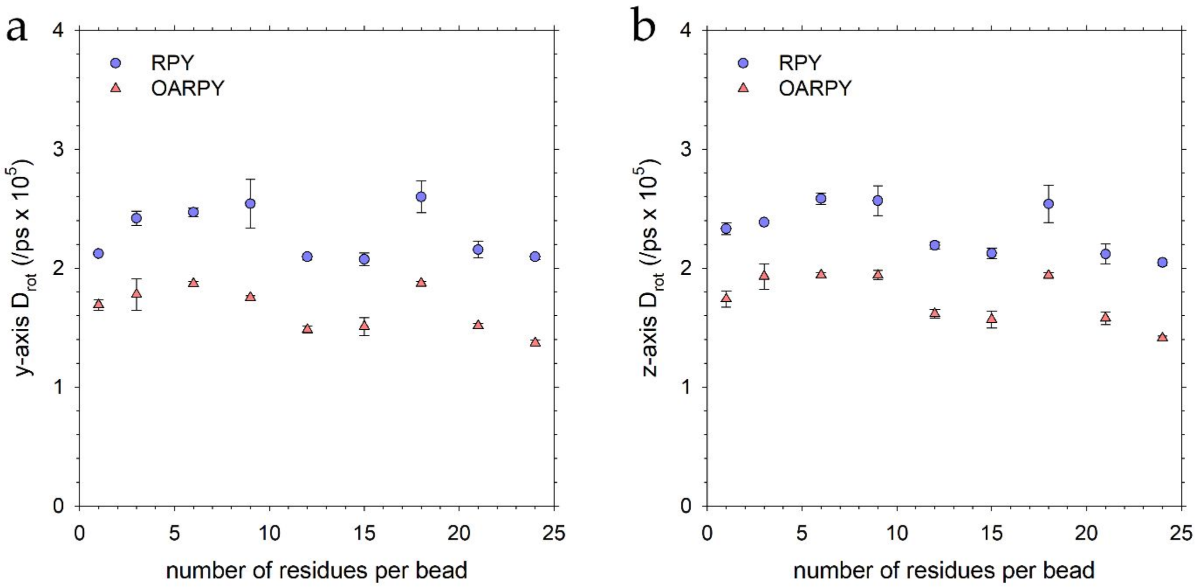

Figure S3 (a) Same as Figure 5c of the main text but showing the rotational diffusion coefficient (D_{rot}) of the principal y-axis of the molecule. (b) Same but showing D_{rot} of the principal z-axis of the molecule.
